# Optimization of Fermentation Condition for Echinacoside Yield Improvement with *Penicillium* sp. H1, an Endophytic Fungus Isolated from *Ligustrum lucidum* Ait Using Response Surface Methodology

**DOI:** 10.3390/molecules23102586

**Published:** 2018-10-10

**Authors:** Fangxue Xu, Hui Cao, Xiaowei Cui, Hong Guo, Chunchao Han

**Affiliations:** School of Pharmacy, Shandong University of Traditional Chinese Medicine, Jinan 250355, China; xfx18364164925@163.com (F.X.); 13553158985@163.com (H.C.); 13553159162@163.com (X.C.); 13589080261@163.com (H.G.)

**Keywords:** Echinacoside, Endophytes, *Ligustrum lucidum* Ait, Response surface methodology

## Abstract

(1) Background: Application of echinacoside has become increasingly important for its significant biological activities. However, there are many disadvantages in existing synthesis methods such as contaminating the environment, harsh reaction conditions and so on. Therefore, it is urgent to invent a novel alternative method that can increase the yield of echinacoside. (2) Methods: In this study, we isolated and purified an endophyte from the leaves of *Ligustrum lucidum* Ait. Then, we improved the yield of echinacoside by optimizing the fermentation condition with an endophytic fungus. *Penicillium* sp. H1 was isolated from *Ligustrum lucidum* Ait. In addition, response surface methodology was used to optimize the fermentation condition. (3) Results: The results indicate that the maximal yield of echinacoside (37.16 mg/L) was obtained when inoculation rate, temperature and days were 13.98%, 27.85 °C and 26.06 days, respectively. The yield of echinacoside was 150.47 times higher under the optimal conditions than under the control conditions. The results indicate that the yield of echinacoside could be improved with endophytic fermentation by optimizing the fermentation condition. We provide an alternative method for echinacoside production by endophytic fermentation in this paper. It may have a profound effect on the application of echinacoside.

## 1. Introduction

**Figure 1:**
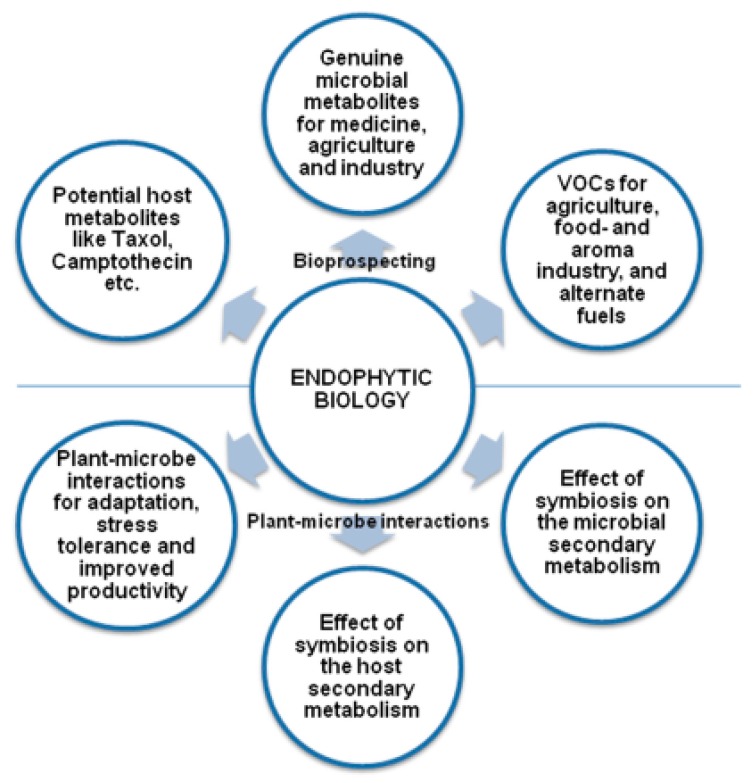
The biological study of endophytes.

Endophytes are non-pathogenic bacteria and fungi that grow intra- or extracellularly in healthy plant tissues such as bark, flowers, roots, stems, leaves and seeds [1,2,3]. There are two transmission routes by which endophytes can be obtained: horizontally from the environment via soil, atmosphere and insects; or vertically from generation to generation via seeds and pollen [4]. Wani et al. [5] summarized that endophytic biology has been studied in numerous targets which can be divided into two categories: bio-prospecting and plant–microbe symbiosis (Figure 1). Endophytes can be used in several industrial branches such as medicine, biotechnology, agriculture, and industrial manufacturing of antimicrobials, insecticides and anti-cancer agents [6,7,8]. It is well known that endophytes can improve plant productivity, stress tolerance and promote phytoremediation [9]. Xu et al. [10] summarized that endophytes could promote the production of rare pharmaceutical metabolites by bio-process optimization strategies. Hence, we studied the technology for isolating and purifying endophytes from plant tissues. In addition, we utilized endophytic fermentation to promote the important component in the plant.

**Figure 2:**
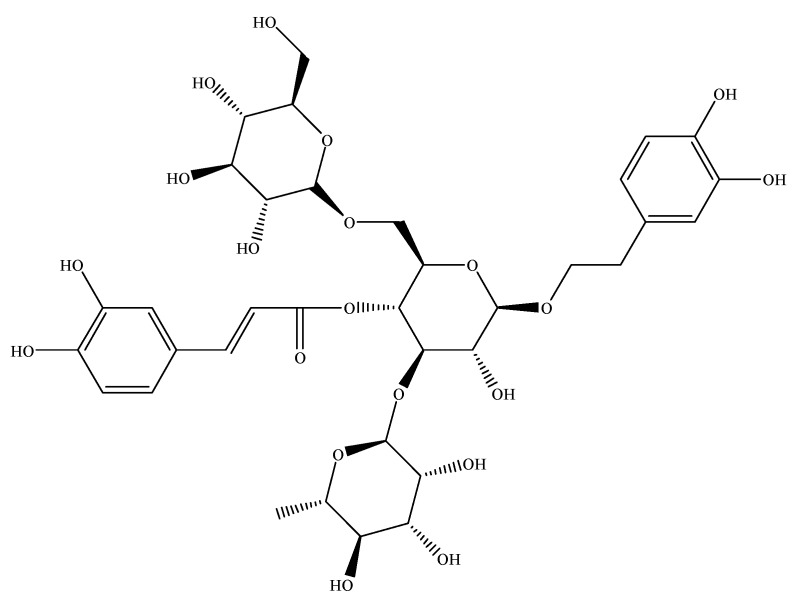
Chemical structure of ECH.

Echinacoside (ECH) is a natural phenylethanoid glycoside extracted from the medicinal Chinese herb *Ligustrum lucidum* Ait. Figure 2 shows the chemical structure of ECH. It resides also in several other plants such as *Cistanche tubulosa* (Schrenk) Wight and *Echinacea angustifolia* [11]. ECH has many valid pharmacological effects such as anti-hyperglycemic [12], anti-inflammatory [13,14], antiosteoporotic [15,16,17], photoprotective [18,19], liver protection [20,21,22], anti-Alzheimer’s disease [23,24,25,26,27], protection of testis and sperm injury [28,29], anticancer [30,31,32] and so on. Although ECH has a wide range of medical uses and familiarities, the amount extracted from plants is low. In general, the concentration of the extracted ECH can be improved by complicated chemical purification process. However, the chemical purification process has disadvantages such as high cost, environmental pollution and so on. Thus, there is an urgent need to find a novel method for obtaining high purity ECH. In this paper, we used endophytic fermentation to increase the content of ECH. It is a novel alternative method to increase the yield of ECH.

Response surface methodology (RSM) is a collection of statistical and mathematical techniques. RSM is used to obtain the optimal parameters in complex systems by constructing mathematical models and analysis of regression and variance [33,34]. It has been used for optimizing the submerged fermentation medium and culture conditions [35,36]. The statistical model based on the Box–Behnken design (BBD) is one of the response surface design methods [37]. In this study, we isolated and identified endophytes from *L*. *lucidum* Ait. Furthermore, RSM was applied to optimize the fermentation condition for improving the yield of ECH.

## 2. Results and Discussion

### 2.1. Identification of Endophyte A. terreus

**Figure 3:**
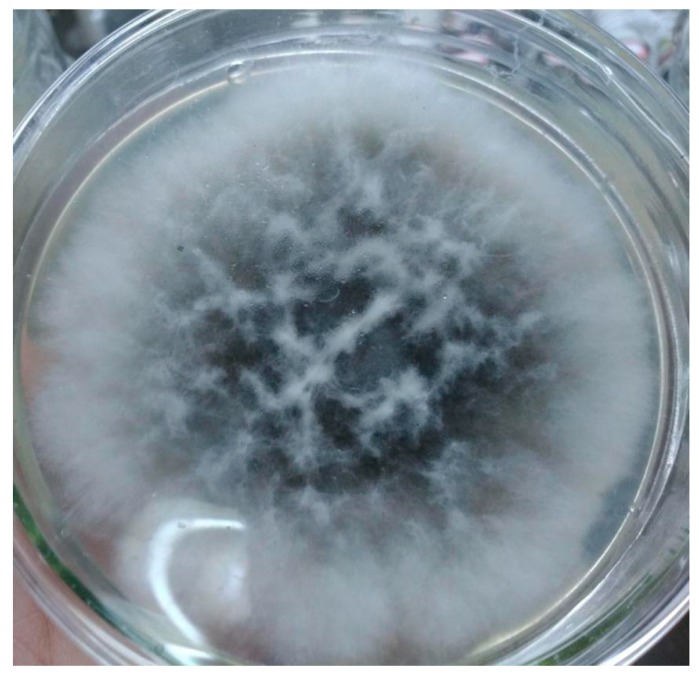
The purified strain in PDA medium.

The purified strain was identified as *Penicillium* sp. H1. The center of the strain colony was green. The edge of the strain was white. The colonial morphology was round. The ITS sequence was BLAST matched with GenBank database on homology, and the similarity with ribosomal rDNA of *Penicillium* sp. was 100%. The purified strain is shown in Figure 3.

### 2.2. HPLC Chromatogram of ECH

**Figure 4:**
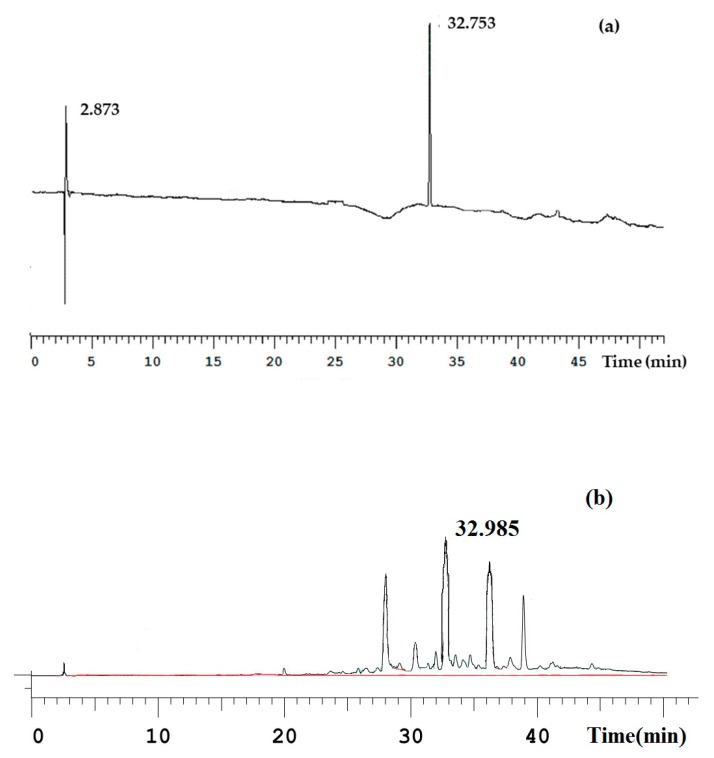
High performance liquid chromatography of: ECH standard (**a**); and sample (**b**). Sample: Fermentation products. The analytical conditions: chromatographic column: ZORBAX Eclipse XDB C18 column (250 × 4.6 mm, 5 μm); mobile phase: acetonitrile (A)−0.1% formic acid solution (B); elution condition: 0–10 min, 7–12% A; 10–35 min, 12–75% A; 35–50 min, 100% A; column temperature: 25 °C; detect wave length: 280 nm; and flow: 1 mL/min.

ECH was eluted at a retention time of 32 min, the same retention time as the reference standard. Figure 4a shows the retention time of the reference standard. Figure 4b shows the maximum ECH yield under the optimal conditions.

### 2.3. Optimizing the Fermentation Conditions by RSM

The BBD was utilized to assess the influence of three individual factors including inoculation rate (X_1_), temperature (X_2_), days (X_3_) and their interaction effects on the yield of ECH. The experimental and predicted values of ECH under different treatment conditions are shown in Table 1. The predicted response Y for ECH production in terms of coded factors is expressed as follow:

Y = 36.98 − 1.76 X_1_ − 0.46 X_2_ + 1.54 X_3_ − 0.42 X_1_X_2_ − 1.99 X_1_X_3_ + 0.50 X_2_X_3_ − 9.62 X_1_^2^ − 10.13 X_2_^2^ − 8.17 X_3_^2^ (1)

where Y is the response variable of ECH, and X_1_, X_2_ and X_3_ are independent variable in coded units. The regression model was statistically detected in the shape of an *F*-test, and ANOVA, which was utilized to evaluate the significance and adequacy of the model (Table 2) [38].

The *p*-value was less than 0.05, demonstrating that model term was significant. As shown in Table 2, the coefficient for X_1_ and X_3_ was significant (*p* < 0.05), indicating that the variables including inoculation rate and days had a significant influence on the yield of ECH. The *F*-value (54.21) suggested that the model was significant. The model was significant with a very low *p-*value < 0.01 (*p*-value Probability > *F*). The values of *R*^2^ (0.9859) and adjusted *R*^2^ (0.9677) were closed to 1, indicating the high efficacy of Equation (1). The value of adequate precision was 17.92, indicating that the model had an adequate signal and could be utilized to navigate the design space. In conclusion, the regression model for ECH yield was a good prediction of the experimental results and the factor effects were real.

**Figure 5:**
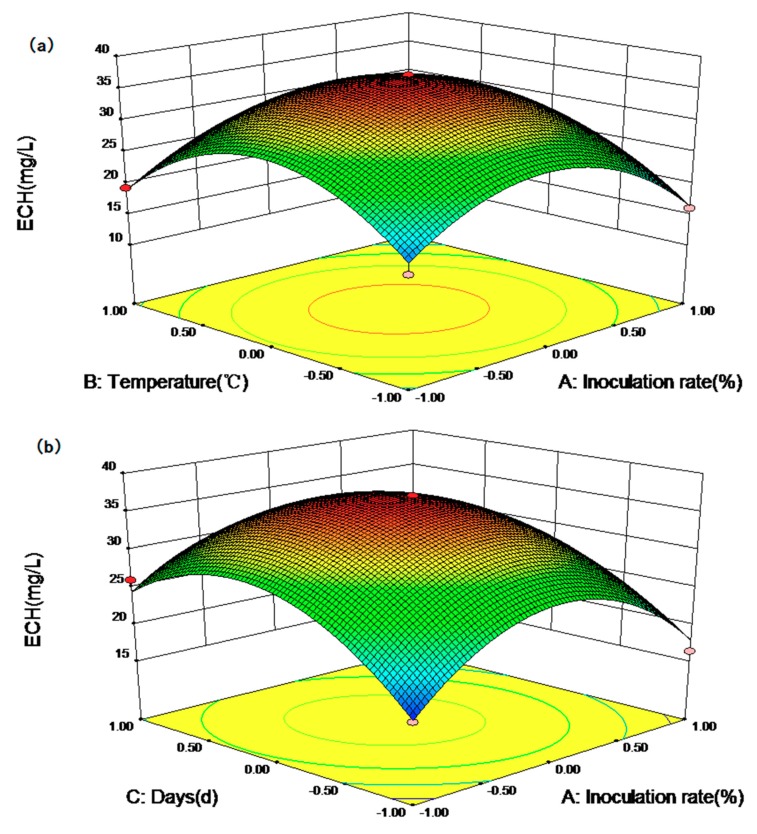

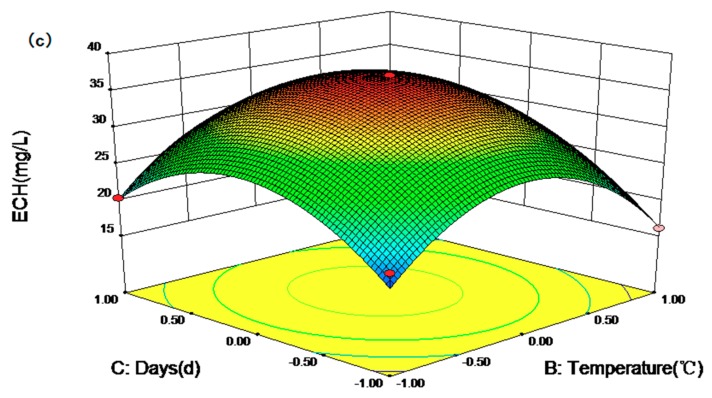
Response surface curves of ECH production: the interaction between inoculation rate and temperature (**a**); the interaction between inoculation rate and days (**b**); and the interaction between temperature and days (**c**).

Figure 5 displays the response surface curves as interaction of inoculation rate, temperature and days on ECH yield. It directly shows the response over a region of independent variables and the relationship between experimental levels of each factor. Figure 5a represents the effects of inoculation rate and temperature on ECH yield, showing that ECH yield increased upon increasing inoculation rate and temperature. A maximal ECH production was obtained when the inoculation rate was 13.98% and temperature was 27.85 °C. Subsequently, the ECH production declined with inoculation rate and temperature increase. Figure 5b shows that increasing days to more than 26.06 days let to decrease of ECH yield. Simultaneously, Figure 5c displays the effects of temperature and days on ECH yield. The trend in Figure 5c is similar to that in Figure 5a,b. Finally, the optimal conditions of inoculation rate, temperature and days were found to be 13.98%, 27.85 °C and 26.06 days, respectively. In addition, the predicted yield of ECH was 37.16 mg/L under the optimal conditions. To ensure the stability of the model equation for predicting the optimal conditions, the experiment was repeated using the optimal conditions. The average result was 37.09 ± 0.11 mg/L. In comparison, the yield of ECH was 150.47 times higher than the control (ethanol extract and water (1:20)).

## 3. Materials and Methods

### 3.1. Reagents and Instruments

The reference standard of ECH was purchased from Shanghai YuanYe Biotechnology Co., Ltd. (Shanghai, China); glucose and peptone were purchased from Beijing AoBoXing Bio-Tech Co. Ltd. (analytical grade, Beijing, China); KH_2_PO_4_ and MgSO_4_·7H_2_O were purchased from Sinopharm Chemical Reagent Co. Ltd. (analytical grade, Shanghai, China); methanol, ethanol, acetonitrile, and formic acid were purchased from Sinopharm Chemical Reagent Co. Ltd. (chromatographic grade, Shanghai, China); and HgCl_2_ was purchased from Tianjin dongfang chemical factory (analytical grade, Tianjin, China).

TDL-5-A centrifuge (ShangHai Anting Scientific Instrument Factory, Shanghai, China); L-2000 Elite high performance liquid chromatograph (Hitachi Limited, Tokyo, Japan); KQ 5200E ultrasound cleaner (Kunshan ultrasonic instrument Co. Ltd., Kunshan, China); LRH-250-Z incubator (medical equipment factory in Guangdong, China); and HX-I incubator shaker (medical equipment factory in Guangdong, China) comprise the used equipment.

### 3.2. Isolation of Endophytes

The leaves of *L. lucidum* Ait were collected from Shandong University of Traditional Chinese Medicine, China in June 2017. Isolation of the endophytes was carried out according to previous methods with minor changes [39]. Leaves were cut into 1 × 1 cm^2^ squares under aseptic conditions. Leaves of *L. lucidum* Ait were immersed in 75% ethanol for 2 min and washed twice with sterile water. Then, the leaves were immersed in 0.1% HgCl_2_ for 3 min and washed three times with sterile water. Finally, the leaves were cultivated in potato dextrose agar medium (PDA: potato 200 g/L, dextrose 20 g/L, agar 20 g/L) at 27 °C for 5 days. The last flush of sterile water as a control was cultured in PDA medium at 37 °C for 5 days. If no microbial growth was found in control, the process of sterilization was successful. The mycelium was transferred into another PDA medium about four times by picking the pure strains for purification. The identification of endophyte was consigned to Shanghai Majorbio Bio-pharm Technology Co., Ltd., Shanghai, China for 16S rDNA sequence analysis and physiological and biochemical characteristics detection.

### 3.3. Preparation of Seed Broth

The acquisition of inoculum was achieved by transferring a few of purified endophyte into seed medium (potato 200 g/L, glucose 20 g/L, KH_2_PO_4_ 0.5 g/L, MgSO_4_·7H_2_O 0.5 g/L) on the rotary shaker at 180 rpm for 7 days.

### 3.4. Ethanol Extract of L. lucidum Ait

First, 70% ethanol 9.5 mL/g and 100 g *L. lucidum* Ait were added to the round bottom flask for water bath reflux extraction over 100 min. The supernatant was obtained by centrifuging for 10 min at 4000 rpm. The supernatant was evaporated to dryness and weighed.

### 3.5. RSM for Optimizing the Fermentation Conditions of ECH

The dry substance of ethanol extract and water (1:20) were used for the culture medium of endophyte. The fermentation culture was carried out on a rotary shaker incubator at 160 rpm. The RSM was used to determine the yield of ECH affected by three factors (inoculation rate, temperature, and days). The yield of ECH was defined as response value. The Box–Behnken factorial design is shown in Table 3. The software Design-Expert 8.0.5.0 trial was utilized in experimental design, data analysis, and quadratic equation construction [40]. Independent variables were coded according to the following formula.

X_i_ = (X_i_ − X_0_/ΔX_i_ (i = 1, 2, 3) (2)

where X_i_ is the coded values of X_i_, X_i_ is the real value of an independent variable, X_0_ is the real value of independent variables on the center point and ΔX_i_ is the step change value [40].

The relationship between variables and response was described according to the following equation:

Y = α_0_ + Σα_i_X_i_ + Σα_ii_X_i_^2^ + Σα_ij_X_i_X_j_ (3)

where Y is predicted response value, α_0_ is the intercept term, α_i_ is liner team, α_ii_ is squared term, α_ij_ is interaction term, and X_i_ and X_j_ are the coded levels of independent variables.

Statistical analysis of the polynomial equation was utilized to assess analysis of variance (ANOVA). The quality of fit of the polynomial model was transmitted by the coefficient of determination *R*^2^, and its statistical significance was measured by the *F*-test in the same program [41].

### 3.6. Analytical Methods

The fermentation broth was centrifuged at 4000× *g* rpm for 20 min to separate mycelia and supernatant. The mycelia and supernatant were evaporated to dryness and weighed. Dry samples were extracted with 50% methanol for 30 min in an ultrasonic bath with 250 W, 40 KHz. The supernatant after extracting was filtered and detected ECH yield using L-2000 Elite high performance liquid chromatograph (HPLC). The analytical conditions: chromatographic column: ZORBAX Eclipse XDB C_18_ column (250 mm × 4.6 mm, 5 μm); mobile phase: acetonitrile (A)—0.1% formic acid solution (B); elution condition: 0–10 min, 7–12% A; 10–35 min, 12–75% A; 35–50 min, 100% A; column temperature: 25 °C; detect wave length: 280 nm; and flow: 1 mL/min.

## 4. Conclusions

In this study, endophyte *Penicillium* sp. H1 was isolated from the leaves of *L. lucidum* Ait. The yield of ECH was highly improved with endophytic fermentation under the optimal conditions, which were inoculation rate, temperature and days of 13.98%, 27.85 °C and 26.06 days, respectively, found using RSM. Due to the extensive pharmacological activities of ECH, the applications are increasing. We provided an alternative method for ECH production by endophytic fermentation in this paper. The yield of ECH was 150.47 times higher under the optimal conditions than under the control conditions. We speculated that ingredients of the medium were converted into ECH by endophytic fermentation. However, the specific mechanism of the biotransformation progress is unknown. In addition, profound studies are required to optimize the culture medium by adding other components such as carbon source, nitrogen source, precursors, ion and so on. Hence, further research is required.

## Figures and Tables

**Table 1:** Design and experimental results of Box–Behnken design.

Standard	Run Order	X_1_	X_2_	X_3_	The Yield of ECH	
					Experimental	Predicted
3	1	−1	1	0	19.31	18.95
13	2	0	0	0	36.98	36.98
8	3	1	0	1	17.21	16.99
7	4	−1	0	1	26.05	24.48
15	5	0	0	0	37.02	36.98
12	6	0	1	1	18.34	20.26
17	7	0	0	0	36.93	36.98
1	8	−1	−1	0	17.34	19.05
9	9	0	−1	−1	20.03	18.11
10	10	0	1	−1	16.04	16.18
4	11	1	1	0	16.31	14.60
5	12	−1	0	−1	17.21	17.43
6	13	1	0	−1	16.31	17.88
16	14	0	0	0	36.91	36.98
11	15	0	−1	1	20.33	20.19
2	16	1	−1	0	16.01	16.36
14	17	0	0	0	37.08	36.98

**Table 2:** ANOVA results of the quadratic model.

Source	Sum of Squares	df	Mean Square	*F*-Value	*p*-Value Probability > *F*
Model	1293.49	9	143.72	54.21	<0.0001
X_1_	24.75	1	24.75	9.33	0.0184
X_2_	1.72	1	1.72	0.65	0.4470
X_3_	19.03	1	19.03	7.18	0.0316
X_1_X_2_	0.70	1	0.70	0.26	0.6239
X_1_X_3_	15.76	1	15.76	5.95	0.0449
X_2_X_3_	1.00	1	1.00	0.38	0.5585
X_1_^2^	389.32	1	389.32	146.85	<0.0001
X_2_^2^	431.71	1	431.71	162.84	<0.0001
X_3_^2^	281.27	1	281.27	106.10	<0.0001
Residual	18.56	7	2.65		
Lack of fit	18.54	3	6.18	1306.47	<0.0001
Pure error	0.019	4	4.730 × 10^−3^		
Cor total	1312.05	16			

*R*^2^ = 0.9859; adjusted *R*^2^ = 0.9677.

**Table 3:** Variables and experiments design level for RSM.

Variables	Coded Symbols	Coded Levels		
		−1	0	1
Inoculation rate (%)	X_1_	5	15	25
Temperature (°C)	X_2_	20	28	36
Days (d)	X_3_	15	25	35
